# STAT3 is required for MiR-17-5p-mediated sensitization to chemotherapy-induced apoptosis in breast cancer cells

**DOI:** 10.18632/oncotarget.15000

**Published:** 2017-02-02

**Authors:** Xing-Hua Liao, Yuan Xiang, Cheng-Xi Yu, Jia-Peng Li, Hui Li, Qi Nie, Peng Hu, Jun Zhou, Tong-Cun Zhang

**Affiliations:** ^1^ Institute of Biology and Medicine, Wuhan University of Science and Technology, Hubei, 430081, P.R. China; ^2^ Key Laboratory of Industrial Fermentation Microbiology, Ministry of Education and Tianjin, College of Biotechnology, Tianjin University of Science and Technology, Tianjin, 300457, P.R. China; ^3^ Wuhan Medical Treatment Center, Hubei, 430023, P.R. China; ^4^ School of Medicine, Wuhan University of Science and Technology, Wuhan, 430065, P.R. China

**Keywords:** miR-17-5p, STAT3, paclitaxel, apoptosis, breast cancer

## Abstract

Signal transducer and activator of transcription 3 (STAT3) controls cell survival, growth, migration, and invasion. Here, we observed that STAT3 exerted anti-apoptotic effects in breast cancer cells. On the other hand, miR-17-5p induced apoptosis in breast cancer cells, and overexpression of miR-17-5p sensitized MCF-7 cells to paclitaxel-induced apoptosis via STAT3. Overexpression of STAT3 in MCF-7 cells decreased paclitaxel-induced apoptosis, but STAT3 knockout abolished the miR-17-5p-induced increases in apoptosis. Finally, miR-17-5p promoted apoptosis by increasing p53 expression, which was inhibited by STAT3. These results demonstrate a novel pathway via which miR-17-5p inhibits STAT3 and increases p53 expression to promote apoptosis in breast cancer cells.

## INTRODUCTION

Signal transducer and activator of transcription 3 (STAT3) is an important transcription factor [1]. In response to interferon, cytokines, and growth factors, Janus kinases (JAK) phosphorylate STAT3, which then forms homo- or heterodimers that translocate to the cell nucleus and mediate the expression of a variety of genes; STAT3 thus plays a key role in many cellular processes, including cell survival, growth, and apoptosis [2]-5]. Furthermore, STAT3 can act as a tumor suppressor or an oncogenic agent; for example, STAT3 suppresses brain tumor progression via PTEN and promotes progression in various breast cancer types [3, 4].

MicroRNAs (miRNAs) are small (approximately 22 nt) regulatory RNAs that base pair with the 3’ untranslated region (3’UTR) of target genes, ultimately resulting in the degradation of target mRNAs or inhibition of their translation [5]. Several studies have demonstrated that miRNAs play important roles in human cancer [6–10], and miR-17-5p is particularly important in breast cancer. Specifically, the miR-17-5p cluster acts as a tumor suppressor by directly inhibiting the expression of *AIB1* and *cyclin D1* in human breast cancer [11, 12]. Yu *et al*. demonstrated that the miR-17-5p cluster mediates migration and invasion in breast cancer cells by affecting the secretion of a heterotypic signal [13]. MiR-17-5p also suppresses MDA-MB-231 cell migration and invasion by inhibiting HBP1 [14, 15]. In addition, miR-17/20 regulates p53 and inhibits Akt, in turn mediating breast cancer cell apoptosis [16], and miR-17*92 increases apoptosis by inhibiting the transition of pro-B into pre-B [17]. The onset and progression of breast cancer involves apoptotic signals that are stimulated by miRNAs [17]. Here we investigated the role of miR-17-5p, which may repress the translation of the *STAT3* oncogene, in the control of breast cancer cell apoptosis.

## RESULTS

### MiR-17-5p sensitized breast cancer cells to stress signal-induced apoptosis

**Figure 1:**
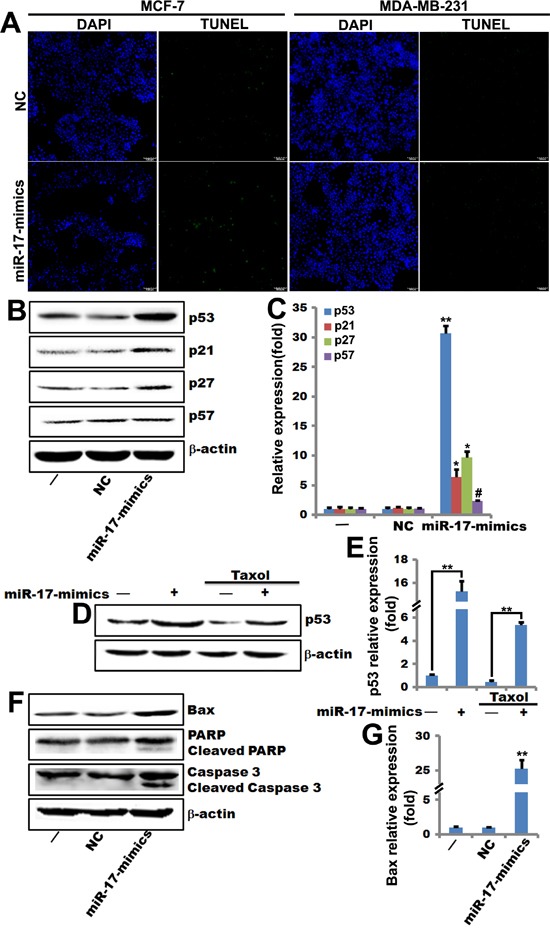
miR-17-5p increases p53 expression and sensitizes breast cancer cells to paclitaxel-induced apoptosis **A**. TUNEL assays using miR-17-5p mimics- and negative control-transfected MCF-7 and MDA-MB-231 cells that were treatment with paclitaxel (0.1 μM) for 48 hours. **B**. and **C**. Western blots (including quantifications performed with Quantity One software) showing p53, p21^Cip1/Waf1^, p27^KIP1^, and p57 levels in MCF-7 cells transfected with miR-17-5p mimics- or negative control. β-actin served as loading control. **, *p*<0.01, *, *p*<0.05, ^#^, *p*>0.05. n=3. **D**. and **E**. Western blots (including quantifications performed with Quantity One software) showing increases in p53 expression in miR-17-5p mimics-transfected MCF-7 cells in the presence (lanes 3 and 4) or absence (lanes 1 and 2) of paclitaxel. β-actin served as loading control. **, *p*<0.01. n=3. **F**. and **G**. Western blots (including quantifications performed with Quantity One software) showing increased Bax, PARP, cleaved PARP, caspase 3, and cleaved caspase 3 levels in miR-17-5p mimics-transfected MCF-7 cells. β-actin served as loading control. **, *p*<0.01. n=3.

Our previous studies demonstrated that miR-17-5p suppressed proliferation in MCF-7 breast cancer cells [18]. To determine the mechanism by which miR-17-5p regulates breast cancer cell apoptosis, MCF-7 cells and MDA-MB-231 cells were transfected with miR-17-5p mimics or negative control (NC). The cells were then treated with 0.1 μM paclitaxel or Taxol for 48 hours and the TUNEL assay was used to analyze cell apoptosis. Transfection of miR-17-5p mimics increased numbers of apoptotic cells in both MDA-MB-231 and MCF-7 cells compared to control cells; this increase was greatest in the MCF-7 cells (Figure 1A). Thus, miR-17-5p strongly increased the sensitivity of MCF-7 cells to Taxol-induced DNA damage.

Next, we performed western blots to measure the expression of target genes (p53, p21^Cip1/Waf1^, p27^Kip1^, and p57^Kip2^) in apoptosis-regulating pathways. P53, p21^Cip1/Waf1^, and p27^Kip1^ expression increased in miR-17-5p-transfected MCF-7 cells. P57^Kip2^ expression was unchanged by miR-17-5p treatment (Figure 1B and 1C). Taxol treatment enhanced the miR-17-5p-induced increase in p53 expression (Figure 1D and 1E). Similarly, expression of the apoptosis gene Bax and cleavage of the PARP and caspase 3 genes increased in MCF-7 cells transfected with miR-17-5p mimics (Figure 1F and 1G). These observations indicate that miR-17-5p sensitized breast cancer cells to stress signal-induced apoptosis.

### MiR-17-5p sensitized MCF-7 cells to tamoxifen

**Figure 2:**
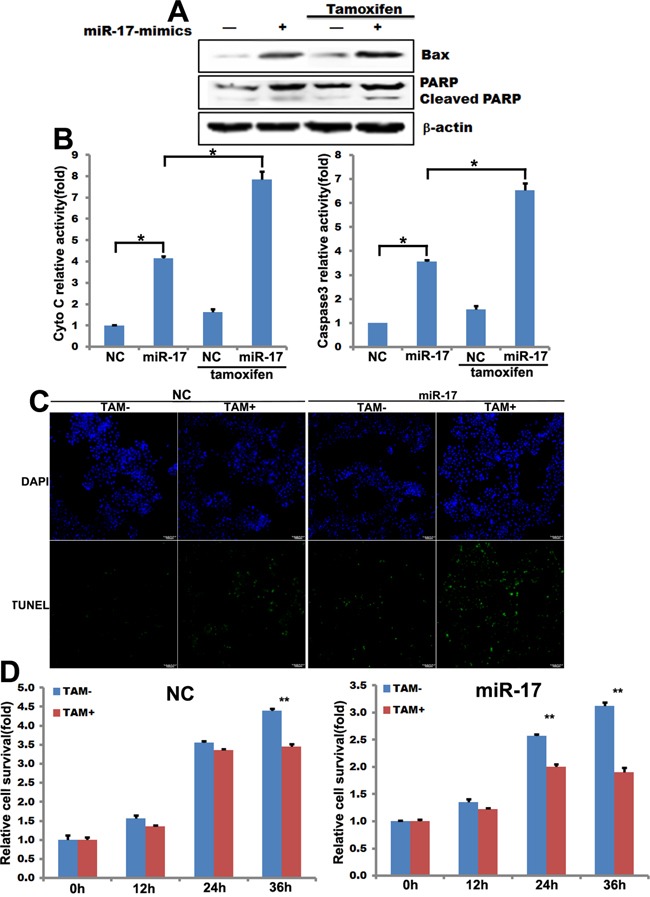
miR-17-5p increases the sensitivity of MCF-7 cells to tamoxifen **A**. Western blot showing changes in levels of apoptosis pathway-related genes in miR-17-5p mimics-transfected MCF-7 cells in the presence (lanes 3 and 4) or absence (lanes 1 and 2) of tamoxifen (15μM) treatment for 36 hours. **B**. ELISA assays showing Cyto C and caspase 3 activity in miR-17-5p mimics-transfected MCF-7 cells in the presence or absence of tamoxifen treatment. The data represent means ± SEM. *, *p*<0.05. n=6. **C**. TUNEL assays with miR-17-5p mimics- or control-transfected MCF-7 cells after treatment 36 hours of treatment with 15μM tamoxifen. **D**. SRB assays measuring the relative survival rate of MCF-7 cells after treatment with tamoxifen (15μM) for the indicated times. The data are shown as means ± SEM. **, *p*<0.01. n=6.

Tamoxifen resistance is common in estrogen-receptor α (ERα)-positive breast cancer cells, including MCF-7 cells [16]. MiR-17-5p-transfected and control MCF-7 cells were treated with 15 μM tamoxifen for up to 36 hours. MiR-17-5p-induced apoptosis in MCF-7 cells was associated with the induction of Bax and PARP and with cleavage of PARP; tamoxifen enhanced this effect (Figure 2A). Similarly, an ELISA revealed that miR-17-5p induced cytochrome c (Cyto C) and caspase 3 expression in MCF-7 cells, and tamoxifen treatment enhanced this effect (Figure 2B). MiR-17-5p mimics-transfected and negative control (NC) MCF-7 cells were then treated with 15 μM tamoxifen for up to 36 hours, and a TUNEL assay was conducted to analyze cell apoptosis. Apoptotic cell numbers increased in miR-17-5p mimics-transfected MCF-7 cells compared to control cells (Figure 2C). Importantly, transfection of miR-17-5p mimics sensitized MCF-7 cells to tamoxifen-induced apoptosis (Figure 2C).

The SRB assay was used to test relative cell survival. Overexpression of miR-17-5p attenuated cell survival in the presence of tamoxifen (Figure 2D). Survival decreased in both control and miR-17-5p-transfected MCF-7 cells after treatment with 15 μM tamoxifen for 36 and 24 hours, respectively, compared to untreated cells (Figure 2D). Furthermore, survival was lower in miR-17-5p-transfected cells at both the 24 and 36 hour timepoints than in control MCF-7 cells after tamoxifen treatment (Figure 2D).

### MiR-17-5p attenuated Taxol resistance in MCF-7 cells

**Figure 3:**
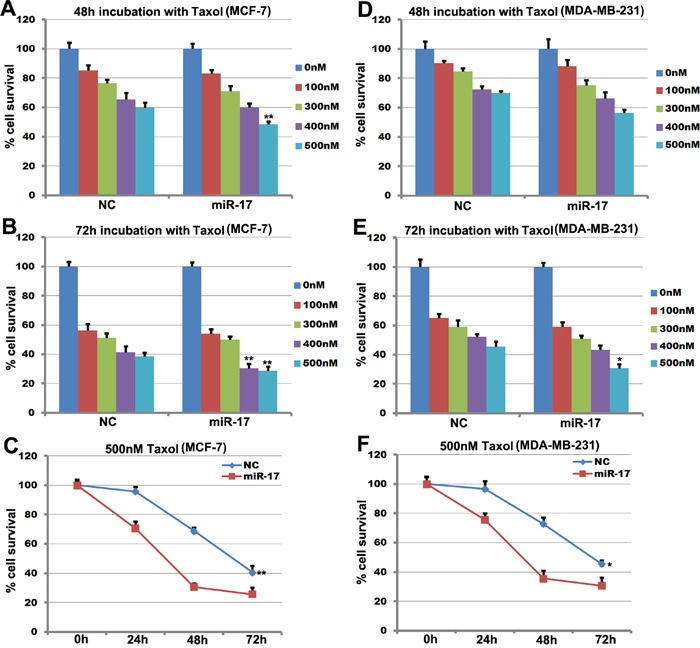
miR-17-5p increases the sensitivity of MCF-7 cells to paclitaxel **A**. and **B**. Survival of miR-17-5p- or control-transfected MCF-7 cells after treatment with paclitaxel at the indicated concentrations for 48 hours or 72 hours. **C**. Cell survival curves for miR-17- and control-transfected MCF-7 cells treated with 500 nM paclitaxel for 0, 24, 48, or 72 hours. The data represent means ± SEM (n=6), **p<0.01. **D**. and **E**. Survival of miR-17-5p- and control-transfected MDA-MB-231 cells after treatment with paclitaxel at the indicated concentrations for 48 hours or 72 hours. **F**. Cell survival curves for miR-17- and control-transfected MDA-MB-231 cells treated with 500 nM paclitaxel for 0, 24, 48, or 72 hours. The data represent means ± SEM. **, *p*<0.01. n=6.

MCF-7 and MDA-MB-231 cells treated with different concentrations (0-500 nM) of Taxol for 48 hours or 72 hours were used for quantitative analysis of cell survival. Survival decreased in miR-17-5p-transfected MCF-7 cells compared to control cells after treatment with 400 or 500 nM Taxol (Figure 3A and 3B). Survival also decreased in miR-17-5p-transfected cells (~29% vs. ~39%) after treatment with Taxol for 72 hours (Figure 3B). MiR-17-5p overexpression reduced the IC50 for Taxol after 48 hours of treatment in MCF-7 cells, and survival decreased in miR-17-5p-transfected MDA-MB-231 cells after 72 hours of treatment with 500 nM Taxol (Figure 3E). In contrast, miR-17-5p did not affect the sensitivity of MDA-MB-231 cells to 48 hours of Taxol treatment (Figure 3D). Cell growth curves revealed that sensitivity to 500 nM Taxol increased in miR-17-5p-transfected MCF-7 cells after 24 hours (Figure 3C). Furthermore, while transfection of miR-17-5p also increased the sensitivity of MDA-MCB-231 cells to 24 hours of treatment with 500 nM Taxol, this effect was weaker than that observed in miR-17-5p-transfected MCF-7 cells in response to the same treatment (Figure 3F). Taken together, these data indicate that miR-17-5p attenuated resistance to Taxol in breast cancer cell lines.

### STAT3 is required for miR-17-5p-induced sensitization of breast tumor cells to Taxol-induced apoptosis

**Figure 4:**
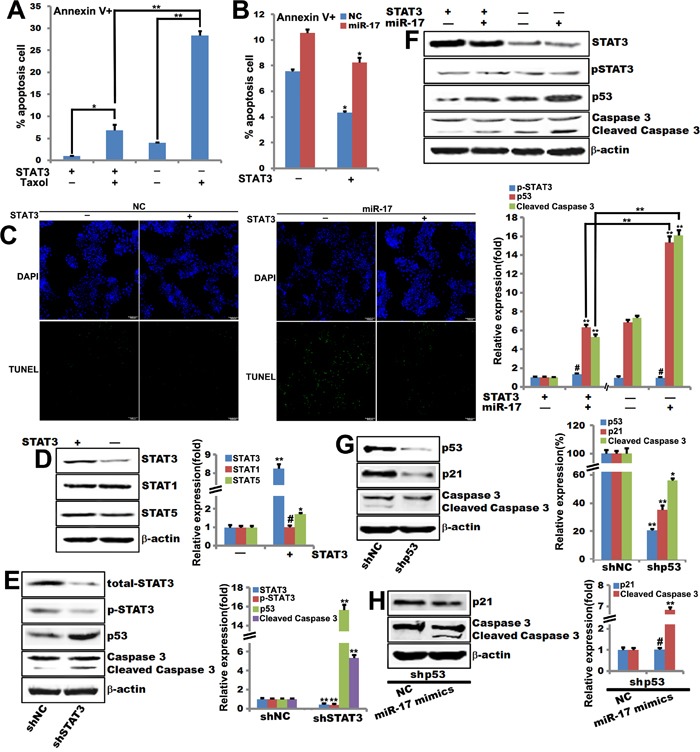
STAT3 is required for miR-17-5p-induced sensitization of breast tumor cells to paclitaxel-induced apoptosis **A**. Annexin V staining of STAT3-overexpressing MCF-7 cells treated with 0.1μM paclitaxel for 48 hours. The data are shown as means ± SEM. **, *p*<0.01. n=6. **B**. Annexin V staining of miR-17-5p mimics- or NC-transfected STAT3-overexpressing MCF-7 cells. The data are shown as means ± SEM. *, *p*<0.05. n=6. **C**. TUNEL assays for detecting apoptosis in miR-17-5p mimics- or NC-transfected STAT3-overexpressing MCF-7 cells. **D**. Western blot of STAT1, STAT3, and STAT5 expression in STAT3-overexpressing MCF-7 cells. Data were quantified using Quantity One software. β-actin served as loading control. **, *p*<0.01, ^#^, *p*>0.05. n=3. **E**. Western blot of STAT3, p53, caspase 3, and cleaved caspase 3 expression in STAT3-knockout MCF-7 cells. Data were quantified using Quantity One software. β-actin is the loading control. **, *p*<0.01. n=3. **F**. Western blot of STAT3, pSTAT3, p53, caspase 3, and cleaved caspase 3 expression in miR-17-5p mimics- or NC-transfected STAT3-overexpressing MCF-7 cells. Data were quantified using Quantity One software. β-actin is the loading control. **, *p*<0.01, ^#^, *p*>0.05. n=3. **G**. Western blot of p53, p21, caspase 3, and cleaved caspase 3 expression in p53-knockout MCF-7 cells. Data were quantified using Quantity One software. β-actin is the loading control. **, *p*<0.01, *, *p*<0.05. n=3. **H**. Western blot of p21, caspase 3, and cleaved caspase 3 expression in p53-knockout MCF-7 cells. Data were quantified using Quantity One software. β-actin is the loading control. **, *p*<0.01, ^#^, *p*>0.05. n=3.

STAT3 inhibits apoptosis by upregulating the transcription of anti-apoptotic genes [19], and JAK2/STAT3 signaling regulates p53 activation [20]. In addition, miR-17 and miR-20a inhibit the expression of p21^Cip1/Waf1^ and STAT3 [21]. To investigate whether STAT3 is involved in miR-17-5p-induced sensitization to cellular apoptosis, we constructed MCF-7 cells with either stable STAT3 knockout or overexpression to assess the role of STAT3 in miR-17-5p mediated apoptosis. These MCF-7 cell lines were transfected with either miR-17-5p mimics or NC. Apoptosis rates were higher in control MCF-7 cells after Taxol treatment than in STAT3-overexpressing MCF-7 (Figure 4A).

MiR-17-5p transduction sensitized STAT3-overexpressing MCF-7 cells to Taxol treatment (Figure 4B and 4C). Notably, p53 expression was much higher in STAT3-knockout MCF-7 cells than in control MCF-7 cells (Figure 4E). Interestingly, expression of STAT5, but not STAT1, was detected in STAT3-knockout MCF-7 cells compared to control MCF-7 cells (Figure 4D). Western blots showed that miR-17-5p overexpression enhanced p53 expression in STAT3-overexpressing MCF-7 cells compared to control MCF-7 cells (Figure 4F).

It has been reported that p53, which is a tumor suppressor, causes cell-cycle arrest and apoptosis in response to DNA damage [22]. Low concentrations of paclitaxel induce p53 and p21 expression and cytotoxic G1/G2 arrest in specific cell types [23, 24]. P53 knockout in MCF-7 cells inhibited p21^Cip1/Waf1^ and cleaved caspase 3 expression compared to control MCF-7 cells (Figure 4G). Interestingly, western blot data showed that miR-17-5p increased cleaved caspase 3 expression in p53-knockout MCF-7 cells compared to control MCF-7 cells; p53 knockout did not affect p21^Cip1/Waf1^ expression (Figure 4H).

### MiR-17-5p directly targeted STAT3 to inhibit its expression in MCF-7 cells

**Figure 5:**
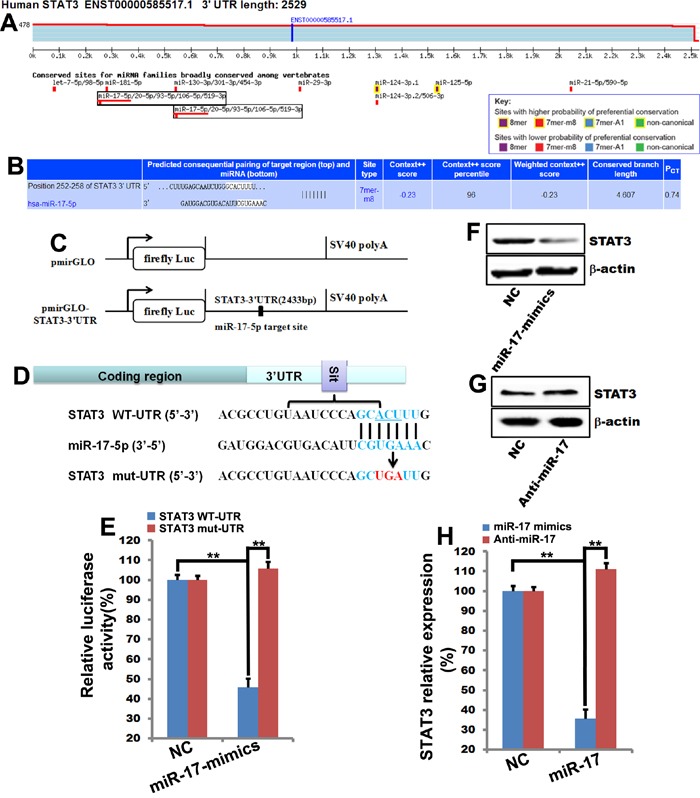
MiR-17-5p directly binds to STAT3 to inhibit its expression **A**. and **B**. Putative miR-17-5p target sites were identified in the 3’-UTR of STAT3 using TargetScan Release 6.2. Bioinformatics analysis revealed two miR-17-5p binding sites in the STAT3 3’-UTR. **C**. and **D**. A luciferase reporter construct containing the STAT3 3’-UTR and the miR-17-5p binding sites in STAT3 3’-UTR. **E**. MCF-7 cells were transfected with STAT3 WT-UTR (STAT3 3’-UTR promoter) or STAT3 mut-UTR (STAT3 3’-UTR promoter with mutated miR-17-5p binding sites) together with increasing amounts of miR-17-5p mimics or NC. Luciferase activity was analyzed. The data represent means ± SEM. **, *p*<0.01. n=6. **F**., **G**. and **H**. 48 hours after MCF-7 cells were transfected with miR-17-5p mimics and miR-17-5p inhibitor (Anti-miR-17), STAT3 expression was analyzed by western blot and quantified using Quantity One software. β-actin was used as the loading control. **, *p*<0.01, ^#^, *p*>0.05. n=3.

To further explore the role of STAT3 in miR-17-5p-induced sensitization to cellular apoptosis, we examined the effects of miR-17-5p on STAT3 expression. Two putative miR-17-5p target sites were identified in the STAT3 3’-UTR using TargetScan Release 6.2 software (Figure 5A), indicating that STAT3 is a potential target of miR-17-5p. These target sites in the *Homo sapiens* STAT3 3’-UTR are shown in Figure 5B. To determine whether STAT3 is a direct target of miR-17-5p, reporter vectors containing either the wild-type full-length 3′-UTR (WT-UTR) or the mutant miR-17-5p binding sites were constructed (Figure 5C and 5D). MiR-17-5p reduced the activity of the STAT3 WT-UTR luciferase plasmid by up to 60%, but had no effect on the activity of the STAT3 mut-UTR luciferase plasmid (Figure 5E). Together, these results indicate that miR-17-5p inhibited STAT3 expression by directly targeting STAT3.

To confirm that miR-17-5p directly targets STAT3, miR-17-5p mimics or anti-miR-17 and the corresponding NC were transfected into MCF-7 cells. MiR-17-5p transfection decreased STAT3 protein levels (Figure 5F and 5H), and anti-miR-17 reversed the miR-17-5p-induced inhibition of STAT3 expression (Figure 5G and 5H). These results confirmed that miR-17-5p inhibited STAT3 expression in MCF-7 cells.

### STAT3 and pSTAT3 expression are elevated, while miR-17-5p expression is decreased, in breast cancer tissue

**Figure 6:**
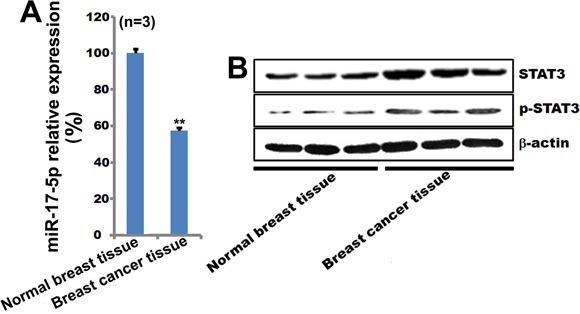
MiR-17-5p, STAT3, and pSTAT3 expression in breast cancer tissue **A**. miR-17-5p expression was examined by qPCR in human breast cancer tissues. U6 was used as a loading control. **p < 0.01. n=3. **B**. STAT3 and pSTAT3 expression were examined by qPCR in human breast cancer tissues. β-actin was used as a loading control.

Since miR-17-5p plays an important role in breast cancer cell apoptosis, we measured miR-17-5p expression in human breast tissues. Compared to normal breast tissue, miR-17-5p expression was lower in breast cancer tissue (Figure 6A). Western blots confirmed that STAT3 and pSTAT3 expression were higher in breast cancer tissue than in normal breast tissue (Figure 6B). These clinical data also indicate that the miR-17-5p-STAT3 axis contributes to the development of breast cancer.

## DISCUSSION

Previous studies have demonstrated that miR-17/20 controls proliferation and induces apoptosis in breast cancer cells [18, 19, 23]. Specifically, miR-17-5p acts as a tumor suppressor by inhibiting cell proliferation in a cell-type-specific manner [18–24]. Active STAT3 controls critical cellular functions, including cell proliferation and differentiation, survival and self-renewal, and apoptosis [25]. The miR-17/92 cluster is a STAT3 target [26], and STAT3-mediated induction of miR-17 expression in particular can confer resistance to MEK inhibitors [27]. These studies provide new insights into the mechanisms by which miRNAs mediate breast cancer cell apoptosis. Here, we report that miR-17-5p-induced sensitization of breast cancer cells to paclitaxel-induced apoptosis requires STAT3.

Tamoxifen exerts its cytotoxic effects primarily through cytostasis, which induces cell cycle arrest at the G0/G1 phase [37]. Tamoxifen also induces apoptotic activity, which involves the cleavage of caspase 3, caspase 7, caspase 9, and poly-ADP-ribose polymerase (PARP) [28, 29]. Importantly, the ER-negative MDA-MB-231, MDA-MB-453, and MDA-MB-468 breast cancer cell lines are sensitive to the cytotoxic effects of tamoxifen [29], and a previous study demonstrated that miR-17/20 enhanced tamoxifen-induced, ERα-mediated apoptosis [30]. This is consistent with our results, which indicate that miR-17-5p increases the sensitivity of MCF-7 cells to tamoxifen (Figure 2).

Paclitaxel (Taxol), a microtubule-targeting agent, can induce G2/M cell cycle arrest and apoptosis [31] and inhibits STAT3 phosphorylation in MDA-MB-468, MDA-MB-231, and MCF-7 cell lines [32]. Taxol attenuates renal interstitial fibroblast activation and interstitial fibrosis by inhibiting STAT3 signaling [33]. Our data indicate that miR-17-5p sensitizes MCF-7 breast cancer cells to Taxol-induced apoptosis (Figure 1) and STAT3 is required for this sensitization (Figure 4).

MiR-17-92 inhibits cell proliferation, promotes apoptosis, and induces tumor angiogenesis [7, 17, 34]. P21^Cip1/Waf1^ is a well-characterized target of miR-17/92 family members, with a 3’ untranslated region (UTR) that is directly regulated by the miR-17/miR-20/mir-106 family [35]. MiR-17-92 overexpression promotes proliferation by inhibiting the expression of p21^Cip1/Waf1^, a cyclin-dependent kinase inhibitor and p53-inducible gene [36]. STAT3 has been shown to bind to the STAT-binding sites (SIE) in the p21^Cip1/Waf1^ promoter, thereby enhancing p21^Cip1/Waf1^ expression [37]. MiR-17 and miR-20a directly inhibit p21^Cip1/Waf1^ and STAT3 expression; conversely, overexpression of both p21^Cip1/Waf1^ and STAT3 reverses miR-17/miR-20a-mediated abrogation of HIF-1α-induced differentiation [21]. P57^Kip2^ is another confirmed miR-17/92 target, and its 3’-UTR harbors binding sites for miR-92 [38]. This may explain why miR-17-5p increased p53, p21^Cip1/Waf1^, and p27^Kip1^ expression, but did not affect p57^Kip2^ (Figure 1B) or p21^Cip1/Waf1^ expression, in stable p53-knockout MCF-7 cells (Figure 4H). MiR-17/20 dampens the reciprocal activation of E2F by c-myc by inhibiting *E2F* translation [39]. MiR-17-5p also inhibits the ERα coactivator AIB1 in breast cancer cells [12]. Rbl2, an Rb family member, is also a target of miR-17-5p [11]. In addition, miR-17/20 targets the *cyclin D1* 3’-UTR in MCF-7 breast cancer cells, resulting in cell cycle arrest and suppression of cell proliferation [11]. Furthermore, miR-17/20 overexpression reduces overall tissue growth in transgenic mice [40]. Here, we found that miR-17-5p induced apoptosis in breast cancer cells (Figure 1A) and increased the expression of p53, Bax, Cyto C, PARP, and caspase 3, which are key components of the p53-mediated apoptosis pathway (Figure 1D, 2A and 2B). Together, these data suggest that the miR-17/20 cluster may function as a breast tumor suppressor by regulating the expression of genes related to cell apoptosis and cell cycle progression.

STAT3, which is constitutively activated in a variety of human tumors, possesses oncogenic potential and anti-apoptotic activities [41, 42]. STAT3 promotes oncogenesis by upregulating the anti-apoptosis genes *Bcl-xL*, *Bcl2*, *Mcl-1*, *survivin*, and *c-Myc* [43–46]. Furthermore, STAT3-regulated miR-17 plays a critical role in MEK inhibitor resistance, and inhibition of miR-17 sensitized resistant cells to AZD6244 by inducing BIM and PARP cleavage [27]. Furthermore, STAT3/miR-17-92 clusters form a positive feedback loop that regulates proliferation of retinoblastoma [16]. Previous studies have revealed that the p53 protein, which acts upstream of p21^Cip1/Waf1^, is induced in response to DNA damage [47]. In addition, Taxol-induced p21^Cip1/Waf1^ and p53 [24, 48] regulate G1/G2 arrest, while low concentrations of Taxol induce mitotic arrest instead [23]. A specific interaction between p53 and a p53-binding site in the proximal region of the miR-17-92 promoter inhibits miR-17-82 expression [49]. A previous study demonstrated that STAT3 binds to the promoter of the mouse *p53* gene to inhibit its expression, and blocking STAT3 up-regulates p53 expression, leading to p53-mediated tumor cell apoptosis [50]. These findings are consistent with the following observations from the current study: miR-17-5p induced apoptosis in breast cancer cells (Figure 1); p53 expression was much higher in STAT3-knockout MCF-7 cells than in the control MCF-7 cells (Figure 4E); miR-17-5p overexpression promoted p53 expression in STAT3-overexpressing MCF-7 cells, but not in control MCF-7 cells (Figure 4F); and miR-17-5p-induced sensitization of breast cancer cells to Taxol-induced apoptosis requires STAT3 (Figure 4).

In conclusion, our study demonstrated that miR-17-5p directly targets STAT3 and induces apoptosis in breast cancer cells by inhibiting the STAT3/p53 pathway. These findings highlight the potential role of miR-17-5p as a prognostic marker and a therapeutic target for breast cancer.

## MATERIALS AND METHODS

### Cell culture and reagents

The MCF-7 and MDA-MB-231 breast cancer cells were cultured in DMEM containing penicillin and streptomycin (100 mg/L) and supplemented with 10% fetal bovine serum (FBS). Tamoxifen (MP Bio) and paclitaxel (Taxol) (Sigma) were used at the doses and for the times indicated in the individual figure legends.

### Oligonucleotides

MiR-17-5p mimics and inhibitors, as well as corresponding negative controls (NC), were chemically synthesized and optimized by Ribobio (Guangzhou, China).

### Quantitative real time RT-PCR for miRNA

Quantitative RT-PCR for mature miR-17 was performed as described previously [51]. The miRNA-specific forward primers from the miScript primer assay (Qiagen, Hilden, Germany) were used. Data are shown as relative expression levels after normalization to U6 (Qiagen).

### Western blot analysis

Whole-cell lysates (50 μg) were separated by 10% SDS-PAGE, and the proteins were transferred to nitrocellulose membrane. The following antibodies were used for Western blots: anti-β-actin (sc-47778), anti-p53 (sc-6243G), anti-p57 (sc-1040), anti-p27 (sc-528), anti-p21 (sc-53393), anti-Bax (sc-7480), anti PARP (sc-7150), and anti-caspase 3 (sc-7148) were from Santa Cruz; anti-STAT1 (#14994), anti-STAT3 (#9139), anti-STAT5 (#9363), and anti-Phospho-Stat3 (Tyr705) (#9145) were from Cell Signaling.

### TUNEL assay

MiR-17-5p-transfected cells and control cells were cultured in medium containing paclitaxel (0.1μM). After 24-48 hours, MCF-7 cells or MDA-MB-231 cells were plated into a 96-well plate in triplicate. Apoptosis assays were performed using the *In Situ* Cell Death Detection Kit, TMR red (Roche Diagnostics, Mannheim, Germany) following the manufacturer's instructions.

### Enzyme-linked immunosorbent assay (ELISA)

ELISA analyses for cleaved caspase 3 (Cell Signaling) and cytochrome c-content (Cyto C) (R&D Systems GmbH, Wiesbaden-Nordenstadt, Germany) were performed according to the manufacturer's instructions.

### Annex V staining

After treatment with Taxol or transfection with miR-17-5p mimics, STAT3-expressing MCF-7 cells were incubated with fluorochrome-conjugated Annexin V and then stained with propidium iodide. Flow cytometry was used to count apoptotic cells.

### Sulphorhodamine B assay

The cytotoxicity of paclitaxel was determined using the sulphorhodamine B (SRB) assay [19]. Cells were plated at 3 × 10^3^ cells/well in sextuplicate in 96-well plates. The cells were then cultured overnight to allow them to adhere, after which culture medium containing different concentrations of paclitaxel was added. Cells were harvested 0 (control) or 72 hours after treatment, fixed with 10% Trichloroacetic acid for 1 hour at 4 °C, and washed five times with water. The cells were then stained with 0.4% SRB in 1% acetic acid for 30 min, washed five times with 1 % acetic acid, and allowed to dry. 10 mM Tris-base was added to dissolve SRB and absorbance was measured with a plate reader at 530 nm. IC50 was defined as the concentration that killed 50% of cells compared to the untreated control.

### miRNA target gene prediction

Genes targeted by hsa-miR-17-5p were predicted using TargetScan Human 6.2 version (released June 2012) (http://www.targetscan.org/).

### Statistical analysis

Data are presented as means ±SEM. The standard two-tailed student's t-test was used for analysis and *p<0.05* was considered significant.
